# Genetic diversity of merozoite surface protein-5 (MSP-5) of *Plasmodium vivax* isolates from Malaria patients in Iran

**DOI:** 10.1186/s12879-023-08804-w

**Published:** 2023-11-17

**Authors:** Sholeh Mansouri, Aliehsan Heidari, Hossein Keshavarz, Parviz Fallah, Amir Bairami, Elaheh Mahmoudi

**Affiliations:** 1 Department of Medical Parasitology and Mycology, School of Medicine, Alborz University of Medical Sciences, Karaj, Iran; 2 Department of Medical Parasitology and Mycology, School of Public Health, Tehran University of Medical Sciences, Tehran, Iran

**Keywords:** Malaria, *Plasmodium vivax*, Merozoite surface protein-5 (MSP-5), Iran

## Abstract

Malaria has not yet been eradicated in Iran, and *Plasmodium vivax* (*P. vivax*) is the main cause of malaria in the country. This study aimed to investigate and analyze the amount of genetic diversity of *Plasmodium vivax* merozoite surface protein-5 (PvMSP-5) exon 1 gene in the southeast of Iran.

Thirty-five patients with clinical symptoms of *P. vivax* malaria participated. The exon 1 of PvMSP-5 was amplified by PCR, and the PCR product of all isolates was sequenced, and genetic polymorphisms were determined using various genetic software.

The analysis showed that studied isolates are different from one another in the DnaSP software version. Out of the 612 sites, 477 were monomorphic and 135 were segregated. The total number of mutations was 143. The singleton variable and the parsimony informative sites were 23 and 112, respectively. There were 17 specific haplotypes with haplotype diversity equal to 0.943. Nucleotide diversity was equal to 0.06766 in the isolates. The ratio of nonsynonymous (0.06446) to synonymous (0.07909) mutations was 0.815020. Tajima’s D, which expressed coding, and non-coding regions, was 0.72403, which was not deemed significant (*P* > 0.10).

The analysis of intrapopulation diversity revealed nucleotide and haplotype diversity in the msp-5 gene of Iranian *P. vivax* isolates. In addition to balancing or purifying selection, intragenic recombination also contributed to the variation observed in exon 1 of PvMSP-5, according to the findings.

## Introduction

Malaria is a significant infectious disease spread by mosquitoes [1]. In 2021, there were approximately 247 million cases of malaria and 619 thousand fatalities worldwide [2]. *Plasmodium vivax* (*P. vivax*) is the predominant cause of malaria in the Eastern Mediterranean region which can cause severe malaria, such as *P. falciparum* [3–5].

Even though, according to the WHO program, Iran is in the phase of eliminating malaria, however, this disease still exists in the country, and malaria transmission is reported to mostly occur in the southeast of Iran, which is categorized as a low transmission area. About 100 cases of malaria are reported in this area every year [6].This area shares a border with Pakistan and Afghanistan. More than 90% of malaria cases in Iran occur in terms of *P. vivax* [7].

The emergence, and development of resistance in *P. vivax* against antimalarial drugs, and the resistance of vector mosquitoes (Anopheles) to insecticides emphasize the need for more efficient methods of malaria management, including vaccine development. merozoite surface protein-5 (MSP-5) is one of the vaccine-candidate antigens of *P. vivax* [8, 9]. A 45KD protein known as *P. vivax* MSP-5 (PvMSP-5) is connected to the microneme or polar capsule of the anterior organelle that participates in merozoite entrance into the host cell. The MSP-5 gene is located on chromosome 4. The gene encoding PvMSP-5 has two exons of about 800 and 300 bp, which are separated by a 400-bp intron [8, 10]. Exon 1 of this gene is the most varied area, and the immune system responds to it as well [10]. In contrast, exon 2 is largely conserved. Exon 1 of PvMSP-5 was amplified and used for genetic diversity analysis as a result. In a study conducted in Indonesia, antibody response to PvMSP-5 was observed in Papuans with acute *P. vivax*, mix-infection with *P. falciparum* and *P. vivax* malaria, and in individuals with a previous history of malaria. In general, the prevalence of IgG response in patients with *P. vivax* was between 42% and 52% [11]. The proteins encoded by *P. falciparum* merozoite surface protein-4 (PfMSP-4) and PfMSP-5 genes have been one of the main groups of antigens considered for malaria vaccines [11]. The nanoparticle vaccine that targeted MSP-4/5 of a *P. yoelii* was highly immunogenic and could provide moderate protection (50–80%) against the blood stage of rodent malaria [12].

*P. vivax*, which is distantly related to *P. falciparum* in the evolutionary tree, was discovered to have homologs of PfMSP-4 and PfMSP-5 [13]. Besides, with the advancement of technology, a new approach was provided to make a vaccine against deadly infectious diseases. As the first mRNA vaccine against severe acute respiratory syndrome coronavirus 2 (SARS-CoV2) showed remarkable success, with the use of this technology, new efforts have started to develop a malaria vaccine. Recently, Hayashi et al. studied the effects of *P. falciparum* circumsporozoite protein (PfCSP) mRNA-lipid nanoparticles (LNP) and Pfs25-mRNA-LNP vaccines against *P. falciparum* in mice. Their research showed that these vaccines, either alone or in combination, may effectively immunize mice against certain *P. falciparum* antigens [14]. However, the diversity of target antigen should be considered, when making a global vaccine against the clinical blood stage of malaria, as it can have a significant impact on the effectiveness of the vaccine [1, 15].

Determining the antigenic diversity of blood-stage genes from the asexual cycle of parasite is one of the main strategies for designing an effective malaria vaccine. Genetic diversity was not observed in PfMSP-5 [16], but it was in a few studies on PvMSP-5 [8, 9].

In Iran, no research on PvMSP-5 polymorphism has been conducted. The purpose of this exploratory investigation was to investigate and analyze the genetic diversity and effectiveness of natural selection in the PvMSP-5 exon 1 gene in Iran.

## Materials and methods

The participants included 40 patients with clinical symptoms of *P. vivax* malaria who were referred to the Health Center of Chabahar, southeast of Iran, in 2020. This number of malaria patients was considered based on the prevalence of disease, and the exploratory nature of the study. After obtaining written consent, 2 ml of blood samples were taken from these patients.

Two competent technicians analyzed the slides generated from the patient’s blood samples under a light microscope for *P. vivax* mono-infection. The steps of DNA extraction were performed using the FavorPrep Blood Genomic DNA Extraction Mini Kit (made in Taiwan) based on the manufacturer’s protocol. Quantitative analysis of the extracted DNA of samples with nanodrop was performed at Alborz University of Medical Sciences. To identify the *Plasmodium* species in the samples, nested PCR was carried out employing ribosomal ribonucleic genes of *Plasmodium* 18 subunit ribosomal ribonucleic (Ssr RNA) genes. The primers and PCR procedure have previously been described [17]. Using the following self-designed order to be made in South Korea by Tekaposist Company, amplification of the exon 1 of PvMSP-5 was performed by PCR on the extracted DNA (Table 1).

Then, PCR was performed at a volume of 25 µl with initial denaturation for 5 min at 94 °C, followed by 35 cycles of denaturation at 94 °C for 45 s, annealing at 59 °C for 1 min, extension at 72 °C for 45 s, and final extension at 72 °C. PCR products were electrophoresed in a 1.5% agarose gel and the bands were observed using a Transluminator.

### Sequence analysis and phylogenetic tree drawing

PCR product of all 40 *P. vivax* isolates was sequenced by Bioneer in Seoul, South Korea. Out of 40 sequenced samples, 35 samples had good readings and five were excluded due to inappropriate readings. The chromatogram of sequences was viewed and edited using Chromas software and EditSeq version 1.7.6. *P. vivax* monoclonal infections were determined by sequencing and using bioinformatics software. Our mono clone sequences were deposited in GenBank as accession numbers: OL449742-Ol449756, Ol4449758-OL449768, and OL396572-OL396580.

Each of the sequences was compared to the sequences in GenBank using the Biological Local Alignment Search Tool (BLAST). The sequenced isolates were aligned by ClustalW software, and genetic diversity was determined by parameters, such as segregating sites (S), the average number of nucleotide differences at each position between two sequences (Pi), the number of haplotypes [18], the average number of pairwise nucleotide differences (K) in the population, and the parsimony of using the same codon, which was calculated using DnaSP version 6.12.03. It was determined that the ENC score ranges from 20 to 61, with values closer to 20 indicating high parsimony and the use of only one codon for each amino acid at each position, and values closer to 60 indicating lower parsimony and the use of all codons for each amino acid [19]. The phylogenetic trees of nucleotide sequences corresponding to the studied isolates, and the sequences received from GenBank were drawn using MEGA version 6.0 under the Windows operating system [20]. The MSP-5 gene of *P. knowlesi* species (Ay573058) as a closely related species to *P. vivax* was used as the outgroup for interspecific comparison with *P. vivax*, as well as for the neutrality tests.

The maximum parsimony method was used to draw the phylogeny tree and compare the sequences [18]. The tree-bisection-regrafting (TBR) algorithm [18] was applied with the default template in MEGA 6.0 software by selecting the external group. 1000 repetitions of validation analysis (bootstrap) were carried out to assure the stability of the branches in the resulting trees. The role of natural selection at the molecular level and the ratio of non-synonymous (dN) to synonymous substitutions (dS) were calculated using the descriptive method [21] in DnaSP software. If dN/dS )ω( is greater than one, it indicates natural selection. If ω is less than one, it illustrates purifying selection or negative selection, and if ω is equal to one, it means neutral evolution [18, 22].

Tajima’s D test [23] and Fu and Li’s D and F tests [24] were used to determine the departure from neutrality.

McDonald and Kreitman (MK) test [25] was used to compare the ratio of nonsynonymous to synonymous changes within and among the species. If the amount of polymorphism within the species is more than the diversity among the species, natural selection is considered. Negative or purifying selection is considered if the quantity of polymorphism within a species is less than the diversity between species. If both are identical, then the hypothesis is null [25, 26].

**Table 1:** The sequence of primers, target gene for amplification of exon 1 Iranian PvMSP-5 isolates

Primers	Sequence	Accession number
Forward	5′- CGC GTC GTG TTA GCT ATC CA-3′	AF403476^a^
Reverse	5′- CAT CGT CTG CCT TGT GTT CG-3′	
Target gene	the exon 1 of PvMSP-5	

^a^The sequence of exon 1 Iranian PvMSP-5 isolates was amplified corresponding to 48-691 PvMSP-5 strain with accession number AF403476 deposited in Genebank

## Results

The analysis of 35 studied isolates showed that the isolates were different from each other in the DnaSP software analysis.

Out of the 612 sites of exon 1 of the Iranian PvMSP-5 isolate, 477 were monomorphic, and 135 were segregate (polymorphic). The total number of mutations was 143. There were 23 singleton variable sites, and there were never three variations of a single site. There were 112 parsimony-informing websites, and eight of them had three variations. Out of the 35 isolates, there were 17 specific haplotypes, and haplotype diversity was 0.943 (Hd: 0.943 variance 0.000032). The average number of nucleotide differences (K) was 41.407.

Nucleotide diversity, which is the average number of nucleotide differences in all studied sites, was 0.06766 in the isolates (Pi = 0.06766). The mutation rate calculated from the polymorphism sites was 0.05674. ENC score was 46.430.

**Fig. 1:**
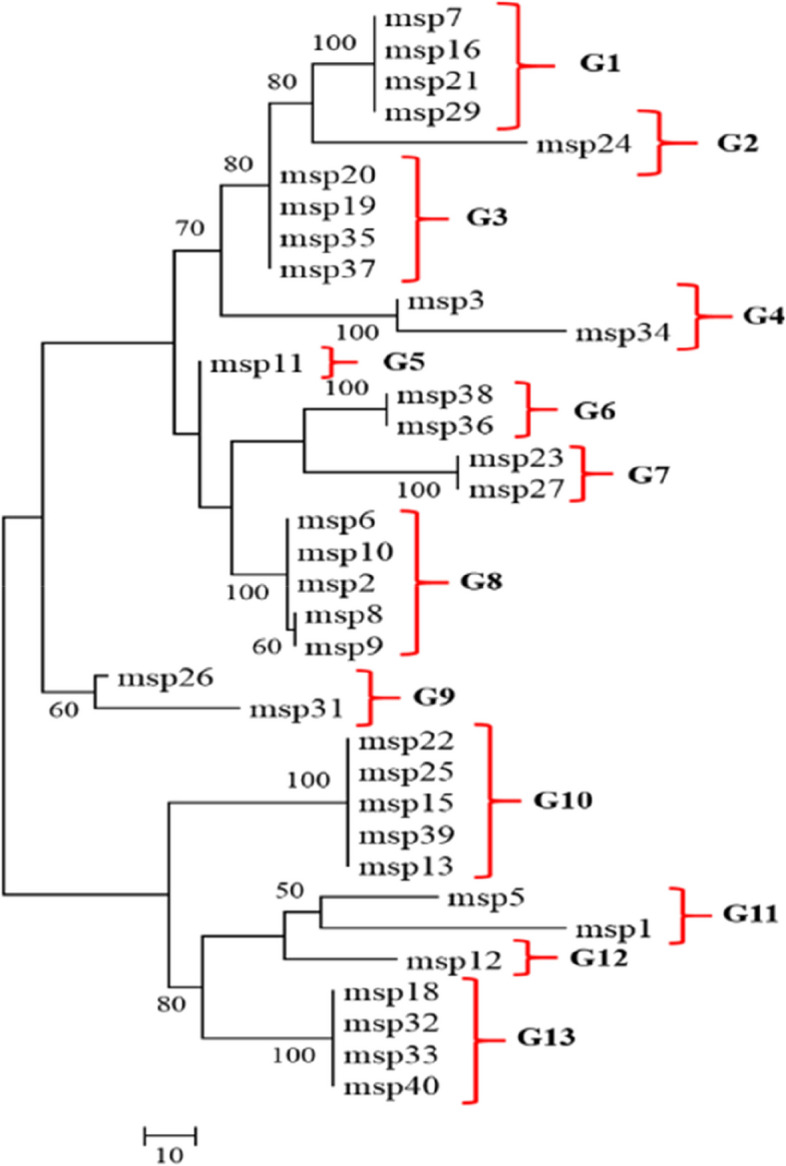
Nucleotide sequence phylogeny of 35 Iranian isolates of PvMSP-5. Phylogeny drew based on the nucleotide sequence of the MSP-5 region and by the Maximum Parsimony method in MEGA ver.6.0 software

**Fig. 2:**
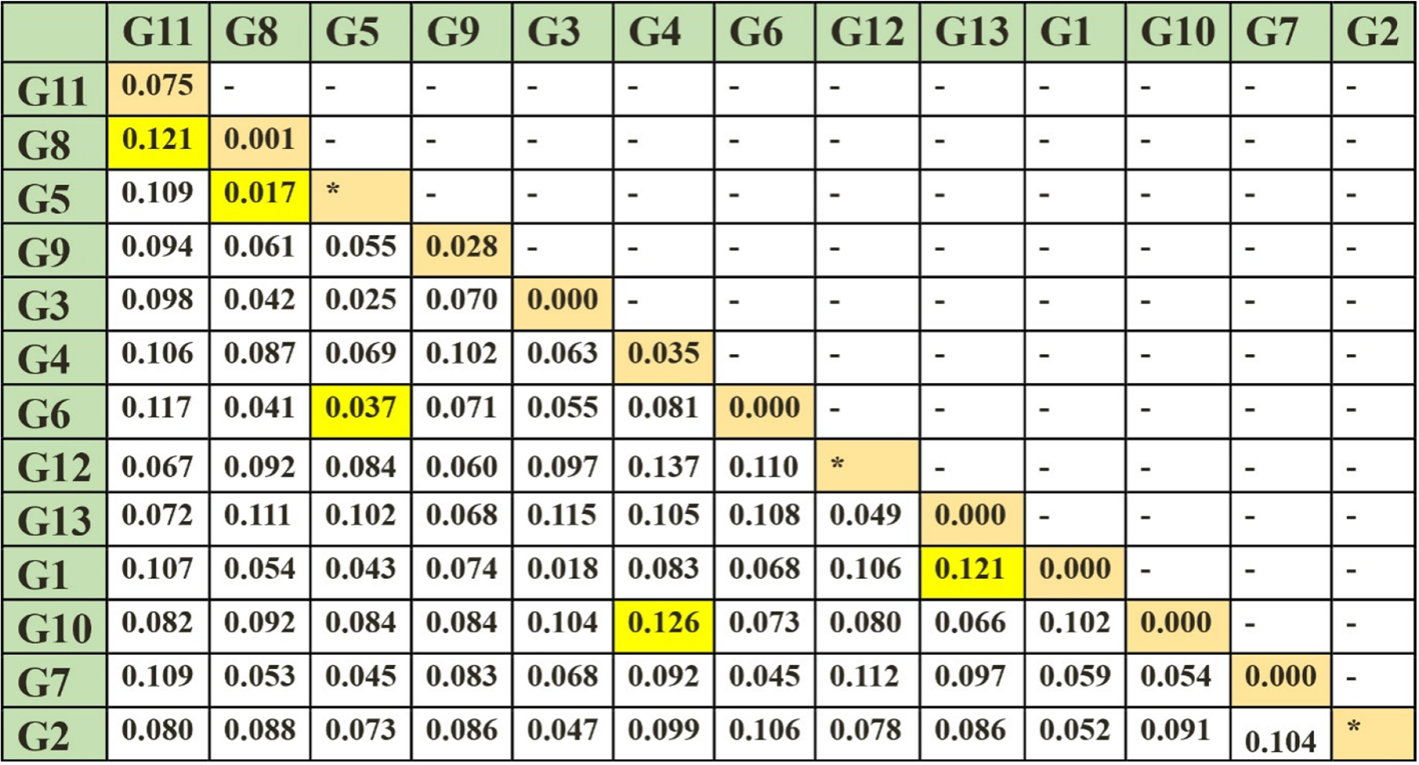
Genetic distance within each group and between different groups was obtained from G1 to G13 by the phylogeny drawn between the Iranian and *P. vivax* isolates

**Fig. 3:**
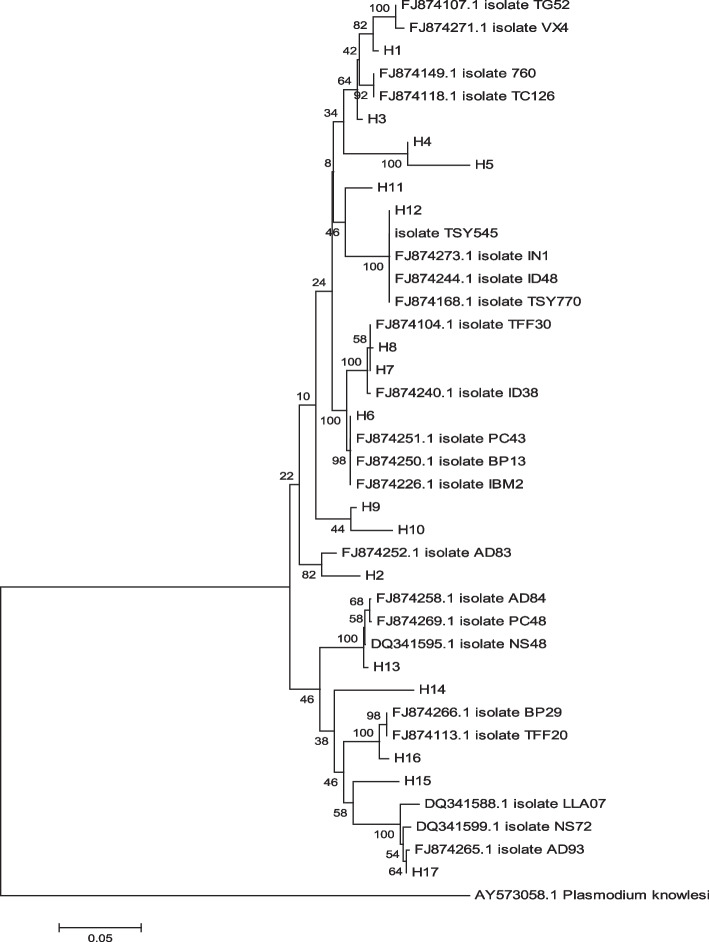
Nucleotide phylogenetic tree of PvMSP-5 gene. Using the neighbor-joining (NJ) method, the tree was drawn with Tamura 3-parameter distance in MEGA version 6.0 software [20]. The numbers on the branches show the percent of bootstrap values according to 1000 replications. The haplotypes reported in our study (H1–H17) with the numbers of isolates recorded in the World Bank Gene are indicated in the tree

Regarding lineage and utilizing the nucleotide maximum parsimony method in MEGA ver.6.0 software, this study observed the placement of various *P. vivax* haplotypes within a single clade, despite minor differences. Consequently, all 35 isolates examined in this study were categorized into 13 distinct clades or groups, specifically labeled as G1 to G13 in the phylogenetic tree (Fig. 1). The genetic distance within each group and among different groups was shown by the phylogeny drawn among the isolates (Fig. 2). Figure 3 shows the similarity of Iranian isolates with the other isolates recorded in GenBank from countries in Southeast Asia and South America.

No nucleotide difference was observed in isolates within groups G1, G7, G10, G6, G3, and G13. The highest nucleotide difference (0.075) was observed among the isolates of the G11 group. The greatest nucleotide difference (0.126) was exhibited between the G10 and G4 groups, followed by the G8 and G11 groups as well as G1 and G13 groups (0.121). Furthermore, the lowest difference between G5 and G8 groups was observed at the value of 0.017, and then between G5 and G6 groups at the value of 0.037.

### Evidence for recombination and natural selection

The ratio of nonsynonymous mutations with dN: 0.06446 to synonymous mutations with dS: 0.07909 is shown in Table 2. Tajima’s D, which expressed coding, and non-coding regions, was not significant (*P* > 0.1) (Table 2).

**Table 2:** Rate of nonsynonymous substitutions using the Nei and Gojobori method [18] with the Jukes and Cantor (JC) correction [27]

dN	dS	dN/dS	Tajima D	Fu and Lis D	Fu and Lis F
0.06446	0.07909	0.815020	0.72403*	0.43379*	0.63514*

*Not significant *P* > 0.10

**Fig. 4:**
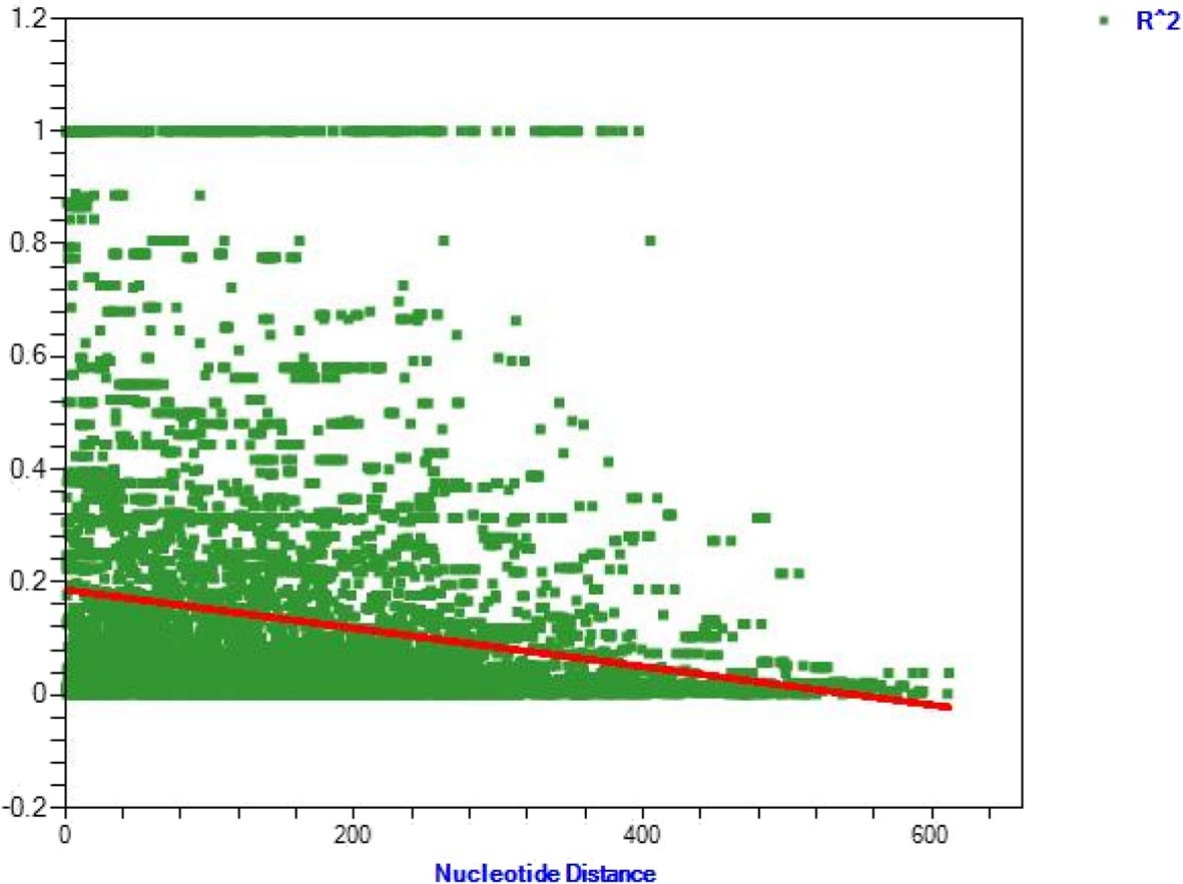
The association between linkage disequilibrium (LD) index (R2), and nucleotide distance among the pairs of sites in 35 Iranian MSP-5 gene of *P. vivax* isolates. The decline of LD index value by increasing nucleotide distance demonstrates that intragenic recombination may appear within Iranian *P. vivax* isolates

Intragenic recombination factors, including Linkage Disequilibrium Rm (minimum number of recombination events), were 22 (Fig. 4).

The statistics for Fu and Lis’ D and Fu and Lis’ F tests were 0.43379 and 0.63514, both deemed statistically non-significant (*P* > 0.10) (Table 2).

Mcdonald and Kreitman’s (MK) test results did not indicate a significant deviation from neutrality, with low rates of intraspecific non-synonymous divergence relative to non-synonymous polymorphisms from *P. knowlesi* as an outgroup species (Table 3).

**Fig. 5:**
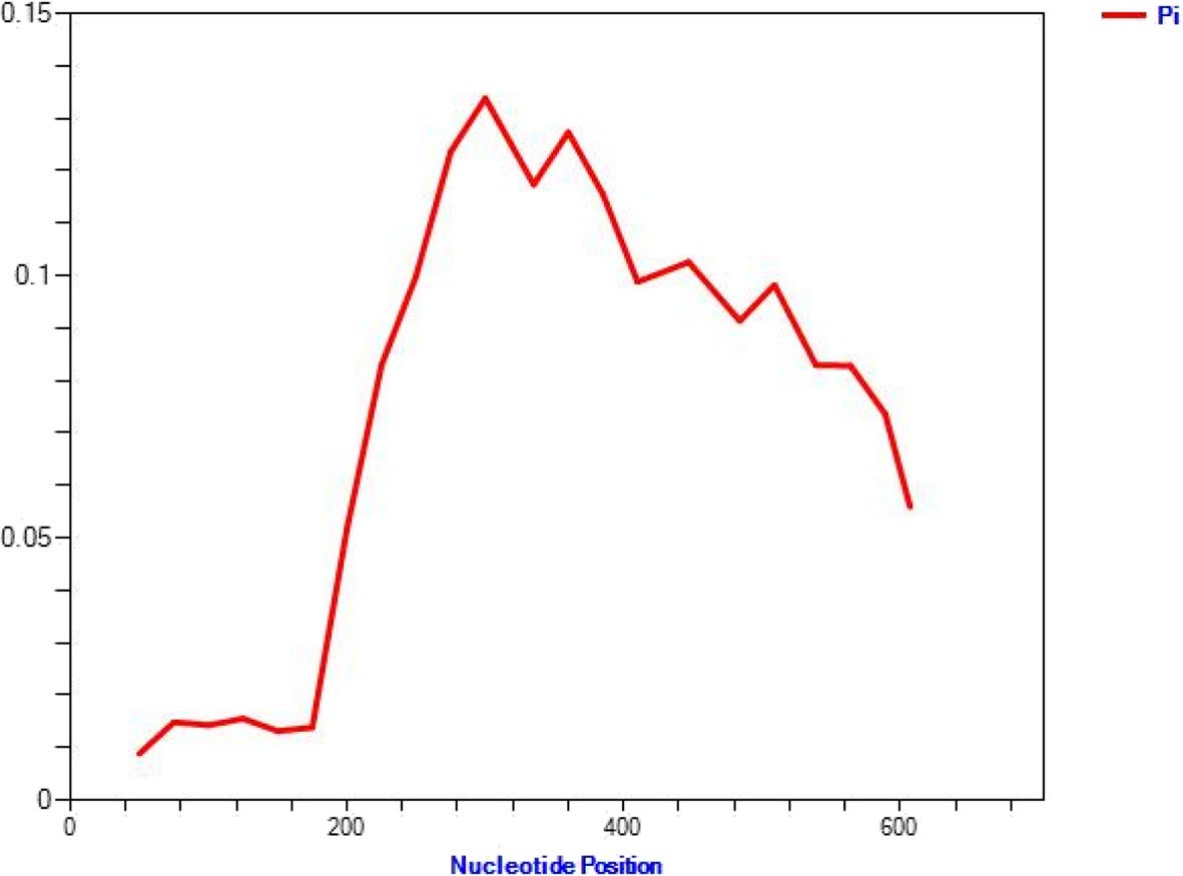
The sliding window plot of nucleotide variation, Pi at the MSP-5 exon 1 in *P. vivax* of Iranian isolates with a window length of 100 bp and a step size of 25 bp. The calculation was conducted using the DnaSP version 6.12.03 software. Maximum diversity was observed among nucleotide sites 226 and 385, as well as 390 and 509 (Significant, 0.001 <* P* < 0.01)

In Fig. 5, a sliding window plot of the nucleotide diversity at MSP-5 exon 1 in *P. vivax* isolates from Iran is shown. The window length is 100 bp, and the step size is 25 bp. The nucleotide positions between 226 and 385, as well as 390 and 509, showed the most variety.

**Table 3:** The findings of the analysis of the McDonald and Kreitman (MK) test. The MSP5 sequence of *P. knowlesi* (Accession No. AY573058) was applied as an outgroup species

Polymorphic changes within species	Fixed differences between species	Neutrality index	Fisher’s exact test (2-tailed)
synonymous nonsynonymous	synonymous nonsynonymous		
40 73	56 126	0.811	0.441 (not significant)

## Discussion

The surface proteins of *P. vivax* merozoites, including MSP-5 can stimulate the human immune system and play an essential role in the invasion of merozoites into the host reticulocytes which are therefore used to make promising vaccines against *P. vivax* malaria [28, 29]. Therefore, in the first preliminary step to consider Iranian *P. vivax* information in designing an effective vaccine against malaria, genetic diversity was determined inthe exon 1 region of the MSP-5 gene of *P. vivax* isolates from symptomatic malaria patients in Iran.

Iranian isolates’ measured exon 1 nucleotide diversity (0.06766) reveals a high degree of genetic variety in the population under study. This degree of variety is roughly in agreement with the findings of a research on *P. vivax*-infected Colombian malaria patients (Pi = 0.049883) [8]. In the study on exon 1 of the MSP-5 gene of 180 malaria patients with *P. vivax* in seven countries, including Thailand, Indonesia, and Brazil, 107 haplotypes were identified, and genetic diversity rates of 0.05014, 0.5747, and 0.05043 were observed in Thailand, Indonesia, and Brazil, respectively [9], which are closer to the genetic diversity of the Iranian isolates in our study. High genetic diversity and recombination were reported in the cited study. The presence of multiple haplotypes, and the high haplotype diversity (Hd: 0.943) further support the notion of a genetically diverse *P. vivax* population in malaria-low endemic areas of Iran. These findings suggest that multiple variants of PvMSP-5 are circulating in the Iranian population.

Considering low-endemic status of malaria in Iran, it was expected that the genetic diversity of exon one would be limited, and the number of haplotypes would be very small. Nonetheless, the proximity of the southeastern region of Iran to Pakistan and Afghanistan, i.e., countries with a high prevalence of malaria caused by *P.vivax*, along with population movements and migration, likely contributes to the introduction of various *P. vivax* variants into the region. This, in turn, increases the genetic diversity of the *P. vivax* population in Iran. Using all of the codons for each amino acid similarly showed that there was little parsimony in the codon selection for each amino acid. ENC score revealed little parsimony between the investigated sequences. The phylogenetic tree analysis constructed from the nucleotide sequences of PvMSP-5 gene shows the diversity, and dispersion among the studied isolates. The presence of distinct genetic lineages, and the divergence of Iranian isolates from other *P. vivax* isolates worldwide suggest the existence of geographically specific genetic variants within the Iranian population. These findings highlight the importance of considering regional genetic diversity when designing malaria control, and vaccine strategies.

Although the value of Tajima’s D was not statistically significant, it was positive (0.72403) in the MSP-5 gene of *P. vivax* in the present study. A positive Tajima’s D indicates balancing selection or population subdivision which shows the presence of a large number of polymorphisms with moderate frequency [23, 30, 31]. The very small sample size in this research might be the cause of the lack of statistical significance. Larger sample numbers in future research might shed more light on the selection factors affecting the PvMSP-5 gene. Moreover, Tajima’s test was not significantly different from zero in the study of Colombian *P. vivax* isolates [8], and in studies on isolates from seven other countries [9].

Fu and Lis’ D and F were positive in the PvMSP5 gene, they are similarly interpreted to Tajima’s D [30, 31] and show a significant amount of moderately frequent polymorphisms. Nonetheless, these two tests did not show significant departures from neutrality, in Iranian isolates, which emphasizes that neutral selection also plays a role in the gene diversity.

The presence of intragenic recombination events, as evidenced by the linkage disequilibrium Rm value, suggests the occurrence of genetic exchange among different PvMSP-5 alleles. Another element that contributes to the creation of novel genetic variants and broadens the gene diversity of PvMSP-5 is intragenic recombination. During the mosquito gut phase of the *P. vivax* life cycle, intragenic recombination takes place [22, 32].The ratio of non-synonymous to synonymous mutations (ω: 0.815) shows that the exon 1 of Iranian PvMSP-5 isolates was not conserved.

If ω is greater than one, it indicates natural selection. If ω is less than one, it shows purifying selection or negative selection [33, 34]. In exon 1, the ω number was higher than 1 in both studies on Colombian and the other seven countries with *P. vivax* isolates [8, 9], but it was 0.815 in our study, and in MEGA software, dS- dN was 0.46; therefore, negative or purifying selection played an essential role in the genetic diversity observed in the MSP5 gene in Iranian isolates. Besides, this suggests that the amount of ω in the MSP-5 gene varies based on geographical area.

Evidence of purifying selection was reported on other merozoite surface proteins, including MSP8 and MSP10 in *P. vivax* [35].

Specific sections of the PvMSP-5 gene that had greater levels of variety were found using the sliding window analysis of nucleotide diversity. These areas could be hotspots for antigenic diversity and need to be investigated further to ascertain their immunological significance and potential as vaccine targets. Although the results of the Mcdonald, and Kreitman (MK) test did not indicate significant deviation from neutrality, the ratio of nonsynonymous to synonymous variation among the species was lower than the ratio of nonsynonymous to synonymous polymorphism within the species (dN/dS < pN/pS). This indicates the presence of negative or purifying selection acting on the PvMSP-5 gene. The results are consistent with the notion that purifying selection acts to remove deleterious mutations, and maintain the functional integrity of the gene.

The presence of distinct genetic lineages and the divergence of Iranian isolates of *P. vivax* from other isolates worldwide have consequences for designing malaria control and vaccine strategies specific to the Iranian population. Different epitope expressions between the varia used in the vaccine and dominant variants in other regions can challenge the effectiveness of the vaccine. Therefore, it is important to consider the specific genetic variants within the Iranian population to optimize vaccine development efforts. Regarding the presence of unique variants in this study, it seems that the dominant variants in this region could be applied to design an effective vaccine. It is suggested that this research be carried out in nearby nations like Pakistan and Afghanistan in order to more accurately outline the identification of PvMSP-5 variations in the area.

The genetic diversity of PvMSP-5 is influenced by two main parameters: purifying selection and intragenic recombination. Purifying selection functions to maintain the functional integrity of PvMSP-5. On the other hand, intragenic recombination contributes to the genetic variability observed within the parasite population. These processes are crucial in the evolutionary and adaptive mechanisms of the parasite, and their interaction has significant implications for vaccine development.

PvMSP-5 demonstrates substantial genetic diversity in and between geographical regions where *P. vivax* is prevalent. Consequently, the existence of diverse isolates presents challenges in the design of a vaccine that can effectively target all variants. Furthermore, it is essential to comprehend the prevalence and distribution of different PvMSP-5 variants in various regions to develop region-specific vaccines.

## Conclusion

The PvMSP-5 of Iranian isolates has substantial nucleotide, and haplotype diversity, based on the examination of intrapopulation diversity. The observed genetic diversity, together with evidence of purifying or negative selection, suggests that the PvMSP-5 gene is under selective pressure to maintain its functional integrity. The findings suggested that, in addition to purifying selection, the intragenic recombination contributed to the variation observed in exon I of PvMSP-5. These results contribute to our knowledge of the genetic structure and evolution of *P. vivax* populations in Iran and have implications for the design of effective malaria control measures, such as vaccines. Future studies with larger sample sizes and a broader geographic scope are needed to further elucidate the selective pressures and functional significance of observed genetic diversity in the PvMSP-5 gene.

## Data Availability

The sequence of the studied isolates is available in the GenBank database as accession numbers: OL449742-Ol449756, Ol4449758-OL449768, and OL396572-OL396580. Other data will be made available upon e-mail request: heidari@abzums.ac.ir.
